# Our Sayings

**Published:** 1903-01

**Authors:** Charles A. Brackett

**Affiliations:** Newport, R. I.


### OUR SAYINGS.[1]

BY CHARLES A. BRACKETT, D.M.D., NEWPORT, R. I.

It is a very simple paper which I bring before you this morning. If its few commonplaces are such as the older and more experienced among you may generally approve, and if they make any useful or

[1] Read before the Harvard Dental Alumni Association, June 23, 1902.

helpful suggestion to any of the younger and less experienced, its object will have been accomplished.

"Out of the fulness of the heart the mouth speaketh," and speech constitutes so large a part of all communication between human beings, that our sayings are of great consequence. What people say to us interests us, amuses us, instructs us, raises our spirits, stimulates our ambition, rouses our courage, moderates our enthusiasm, excites our anger, inspires and cultivates friendship, makes us miserable or fills us with happiness. What people say of us makes or mars that good name which is rather to be chosen than great riches.

The sayings of every person, from the youngest child whose prattlings give joy to the mother's heart, up through all the gradations of years and experience and through all the affairs and relations of life, are of consequence. Of special importance are the expressions of those to whom others go for any sort of advice or help in any of their concerns. Within this category stands the dentist, and we should always seek to have our expressions to our patients governed by a wise discretion. Those who come to us should always be able to see in us cheerfulness and cheeriness, frankness, sincerity, uprightness, and the desire to serve. Both self-respect and modesty should be apparent. Compliance and firmness have each their place. We should be chary of promises, and yet be possessed of that self-confidence which knows what it can do. Beyond saying hopefully that we will try, there should seldom be advance assurance of what we will do. Let the thing done be the declaration. When we are in fault, as every one must sometimes be, a readiness to acknowledge it and take our share of blame is always the best way out. We would better have our ability questioned than our honesty. Never tell any one that an operation will not hurt when you have reason to believe that it will hurt. Never deceive a child, nor permit any one, parent or otherwise, who accompanies it to make untrue statements to it while it sits in your chair. Distrust in a child from former experience is a most pitiful thing to see; while one cannot fail to have great gratification in the submission and co-operation, under painful procedures, of a good child who has always found that it could rely implicitly upon what it was told.

Few patients come to our chairs without some feelings of trepidation. Often there is reason for dreading our inflictions; but there are many instances in which the apprehension is not well founded. Either the operations will not hurt, or they may be so palliated that they will be quite bearable. Remembering the shrinking which we, ourselves, have from being hurt, we should strive both to make our inflictions upon others as light as thoroughness of work will permit, and to do all that we can to set aside needless apprehension. Before we begin the work and during its progress we may do and say much to disarm apprehension, and to cheer and encourage against timorousness. And we should not only say things, but in our tone and manner we should make it apparent beyond question to the patient that we sincerely sympathize, and that we are not inflicting and will not inflict any hurts that we can reasonably avoid.

The dentist especially needs self-control. He needs it himself for his own sake in order that he may maintain his mental balance and work to the best advantage, and he needs it again because often his control must supply the place of the patient's self-control. Real self-control is, of course, largely a matter of temperament, but it is also largely a matter of cultivation. Numbers of people remarkable for their equanimity and mildness of expression were by nature very different, and they have attained their graces through persistent effort. It is a great help under exasperating circumstances to cultivate the habit of hesitancy of speech, and of speaking in a tone lower, or at least not higher, than usual. One should seek to frame one's sentences so that it cannot be charged by the other party that they contain untruth, inaccuracy, exaggeration, or misrepresentation. It is good practice in self-control never to use exclamations or expletives under any circumstances. Mishaps, accidents, surprises, failures, alarms, shocks, of little or of much consequence, come to us. Under such circumstances many people make exclamatory expressions practically involuntarily. It is with them simply a habit. With a little attention and effort one may form the infinitely better and, for the dentist, far more becoming habit of being silent, whatever happens, until a rational sentence can be formulated.

The dentist should be very careful in all his expressions concerning others. Never let it be said that your office is a good place to get the news. Seldom introduce a personal topic of conversation, and then only to commend something which is good and worthy. Ascribe to others good intent and right motives; make allowances for mistakes, misunderstandings, and misapprehensions. Do not mind trifles. Cultivate a broad-mindedness and a high-mindedness. Observing these things, your judgment of your fellows is more likely to be a just judgment than if you were governed by a meaner spirit. With the right spirit, we shall find but most rarely in the work of any other practitioner anything to condemn or even to pass over in silence; but we shall find all along the way multitudes of instances for commendation.

Do not talk too much. In a particular community a lady of wealth and prominence entered a store. A salesman, who knew her face but was utterly unknown to her, came forward and greeted her effusively. "Good-morning, Mrs. So-and-So. It's a fine morning this morning. I was pleased to see you at the opera last evening." The lady put on her eye-glasses, looked him up and down, and said, "Young man, I came here to buy a carpet."

Some of our patients come to us to have a tooth filled. Undivided diligence in filling the tooth is what is desired. Above all, do not burden others with any of your own cares or trials. Before you say anything question if saying it will serve any good and useful purpose or make anybody really wiser, better, or happier. No one is benefited by having you retail that you did not sleep last night, or that mosquitoes have bitten you, or that you have dyspepsia, or that the cook has left and the plumber did not come. First, do not have troubles; second, when you have troubles, do not mind them; third, when you mind them, do not talk about them.

Do not talk too little. We all of us have many patients whose satisfaction in their calls upon us is much helped by our cheery sociability, many whom we can interest and advise, encourage and comfort. I have just intimated that we should not ordinarily make our own cares subjects of conversation. With equal earnestness I would say that we should be ever ready to listen to the troubles of others, and alert to be as helpful as we can to every one who is really in need of such cheer and comfort as we can give.

A lamentable case of inconsiderate cruelty of expression was under these circumstances; a skilled and careful dental practitioner had been nursing along for some time a case of disease in the mouth which he recognized as malignant. The patient was very ill, but neither he nor any of his friends had knowledge of the grave significance of his malady. If I remember rightly, the patient's wife was of an hysterical temperament and a victim of heart disease, which made anything of a mental shock a great risk for her. One day when the patient and his wife were in the office, the attending practitioner had a call from another dentist whom he politely asked to see the case. At the first glance the visitor blurted out, in a voice to be heard through consulting- and waiting-rooms, "Well, I don't know what you think about it, but I think that is a cancer." The drop had fallen; and the harm done by the inconsiderate expression could never be repaired.

To offset this I may give you some more kindly instances. A gentleman, now past eighty years of age, has for a long time at annual examinations pointed out a buccal filling in a right lower second molar as having been made when he was twenty-six. Really his present dentist refilled the cavity a dozen years ago, but he is quite content that the old time service should have the credit.

Nothing in the way of faith could be more implicit than one elderly lady's confidence in mutton tallow as a panacea for every sort of a toothache. In her case the most intense odontalgia yields at once to a free inunction upon the gum. Some of her friends have hard work to accept and use her remedy; but her dental adviser only expresses rejoicing that she has such a ready and effective resource.

In the beginning of my practice an estimable old lady, long since now of blessed memory, came in wearing a partial lower plate with two ordinary porcelain central incisors attached. She told me of her long-cherished and deep-seated repugnance to wearing artificial teeth, and of how, when her own teeth had dropped out, her dentist had taken them and mounted them on the plate, and she had such satisfaction in wearing them. He would have been a black-hearted wretch who would have destroyed the sweet illusion.

You will note that the writer draws a line of distinction between wilfully deceiving, and permitting others to hold opinions to which they are entitled.

In our interviews with our patients, then, discretion, tact, honesty of purpose, and kindness of heart should govern our sayings.

